# Prevalence and associated factors of e-cigarette use tendency among adolescents in a rural community, Thailand: A cross-sectional study

**DOI:** 10.18332/tid/218288

**Published:** 2026-04-22

**Authors:** Nathan Yinpraphan, Nara Hansang, Nawarit Sukcharoen, Piraya Poommin, Pannawit Srinithiwat, Chonapat Khunlapate, Prime Charoenpitakporn, Ploypun Narindrarangkura

**Affiliations:** 1 Phramongkutklao College of Medicine, Bangkok, Thailand

**Keywords:** adolescents, e-cigarette, risk factors, tendency, Thailand

## Abstract

**INTRODUCTION:**

Adolescent electronic cigarette (e-cigarette) use has increased globally despite regulatory bans. In Thailand, e-cigarettes have been prohibited since 2014; however, adolescent tendency toward e-cigarette use remains a concern. This study aimed to determine the prevalence and associated factors of e-cigarette use tendency among secondary school students in Chachoengsao, Thailand.

**METHODS:**

A descriptive cross-sectional study was conducted during 18–22 January 2025, among 1848 students aged 13–18 years from 19 secondary schools in Chachoengsao. Participants completed an online self-administered questionnaire on demographics, behaviors, social influences, and e-cigarette knowledge. Tendency was defined as self-reported thoughts about using e-cigarettes. Data were analyzed using descriptive statistics and univariate and multivariate logistic regression, with adjusted odds ratios (AORs), 95% confidence intervals (CIs), and p<0.05 indicating significance.

**RESULTS:**

Among 1848 students, 17.6% (n=326) reported a tendency to use e-cigarettes. Being female (AOR=1.66; 95% CI: 1.20–2.30) and low household monthly income (<5000 compared with 20000–29999 THB; AOR=2.81; 95% CI: 1.26–6.25) were associated with a higher tendency. Cigarette smoking was the strongest predictor (AOR=6.71; 95% CI: 4.59–9.82) and alcohol use was also significant (AOR=1.71; 95% CI: 1.16–2.54). Peer influence, including neutral attitudes (AOR=1.42) and prior encouragement (AOR=1.95), increased the likelihood of tendency. Perceived social acceptance (AOR=1.75) and misconceptions – such as believing secondhand vapor is harmless (AOR=3.68) or that e-cigarettes aid smoking cessation (AOR=1.93) – were independently associated with tendency.

**CONCLUSIONS:**

Nearly one in six students tended toward e-cigarette use despite the national ban. Female gender, low household monthly income (<5000 THB), risk behaviors, peer influence, and misconceptions were significantly associated factors. Further longitudinal studies are warranted to clarify causal relationships and provide stronger evidence for targeted interventions.

## INTRODUCTION

Electronic nicotine delivery systems (ENDS, e-cigarettes) initially emerged in China in 2003 and have since become widely available worldwide, particularly through the Internet^1^. By 2018, many countries had enacted national ENDS laws due to growing concerns about health risks and misuse^2^. Despite these regulatory efforts, adolescent use of nicotine products remains a public health concern. Longitudinal evidence suggests that early substance experimentation and tendency during adolescence are associated with progression to regular use and engagement in other risk behaviors^3^. In the United States, 2024 estimates reported use among 3.5% of middle school and 7.8% of high school students^4^. Contributing factors include the easy accessibility of e-cigarettes through online sales and pervasive advertising, with 78.2% of middle and high school students reporting exposure to e-cigarette marketing^5^.

E-cigarettes contain high concentrations of nicotine and multiple harmful chemicals, raising concerns about serious health impacts among adolescents^6-8^. Nicotine levels in many devices exceed those in traditional cigarettes, intensifying the potential for addiction among teenagers^6,7^. Nicotine exposure during adolescence disrupts neurodevelopment, particularly in brain regions responsible for executive function, working memory, learning, and impulse control, and has been associated with mood disorders and impaired behavioral regulation^6^. Beyond nicotine, aerosols contain toxic additives such as propylene glycol, glycerol, flavoring agents, and metals released from heating coils, which elevate respiratory and cardiovascular risks and may cause long-term damage to the developing heart and lungs^6-8^. Misconceptions about safety, limited awareness of health harms, peer pressure, and the appeal of diverse e-liquid flavors further reinforce use among adolescents^9^.

Thailand reflects global trends but presents a paradox. Thailand has implemented comprehensive tobacco control strategies supported by the Thai Health Promotion Foundation, which has played a central role in advancing population-level tobacco control policies and behavioral change initiatives^10^. Nevertheless, adolescent e-cigarette use has increased despite the national prohibition on the importation and sale of e-cigarettes enacted in 2014 and reinforced in subsequent years.Ever use among Thai middle school students increased from 3.3% in 2015 to 7.2% in 2021^11^. However, limited evidence exists regarding contextual drivers of e-cigarette use among Thai adolescents^12,13^. Socio-ecological determinants – including socio-economic status, family dynamics, cultural attitudes, exposure to marketing, and product literacy – have been shown to influence adolescent e-cigarette use; however, findings vary across sociocultural contexts^14^. In jurisdictions with regulatory prohibitions that may constrain reliable self-reporting, tendency measures provide a methodologically appropriate proxy for future uptake.

To address these gaps, the present study focuses on Chachoengsao Province, where prior community-based research conducted in a rural subdistrict has highlighted the influence of socio-ecological factors on adolescent risk behaviors^15^. The province, therefore, provides a relevant setting to explore determinants of youth e-cigarette tendency in similar semi-rural provincial contexts across Thailand. The study aims to determine the prevalence of the tendency to use e-cigarettes and to identify associated factors among secondary school students.

## METHODS

### Study design

A school-based descriptive cross-sectional study was conducted to investigate factors associated with e-cigarette use among students aged 13–18 years in Chachoengsao Province, Thailand (see Supplementary file for the STROBE checklist). Recruitment was coordinated with administrators of all 39 secondary-level educational institutions in the province. This study was approved by the Ethics Committee and Institutional Review Board of the Royal Thai Army (M014q/67). Participation was strictly voluntary, with confidentiality and privacy guaranteed. Written informed consent from parents/guardians, as well as assent from students, was required prior to access to the survey.

### Study setting and population

The study was conducted in 19 of the 39 educational institutions in Chachoengsao Province, comprising 29 public schools, 8 private schools, and 2 municipal schools. Together, these institutions represent an estimated 38721 students aged 13–18 years. A census sampling approach was used to recruit all eligible secondary school students enrolled in participating schools during the study period. Students who did not provide assent or lacked parental/guardian consent were excluded. Schools participated voluntarily; therefore, selection bias cannot be excluded.

### Data collection

Data were collected using a self-administered online questionnaire distributed through participating schools. The instrument included demographic variables: school affiliation (public/private/municipal), district (11 districts in the province; responses obtained from 8 districts), GPA (<2.00 to 3.50–4.00), sex (male, female), age (years), ethnicity (Thai, Other), religion (5 categories), and family monthly income (<5000 to ≥30000 THB).

Potential associated factors comprised parental education (7 levels), parental marital status (6 categories), living arrangement (4 categories), perceived family economic status (low, moderate, wealthy), exposure to smoking, exposure to e-cigarette advertisements, perceived guardian and peer attitudes, admired persons’ e-cigarette use, encouragement to use e-cigarettes, and perceptions regarding stress relief, portability, flavors, accessibility, and social acceptance (categorical). Knowledge items were assessed using true/false/not sure responses.

The dependent variable was the tendency to use e-cigarettes (no tendency vs tendency). Only variables conceptually and empirically relevant to e-cigarette tendency were included in the regression analysis. Knowledge items were analyzed descriptively and were not entered into the multivariate model. Sex, age, GPA, and family monthly income were treated as potential confounders.

The questionnaire was developed based on a review of existing literature and previously published instruments assessing adolescent e-cigarette use and related psychosocial factors. Items were adapted to reflect the Thai sociocultural context. Content validity was evaluated by a panel of three experts in public health, adolescent medicine, and behavioral science, who assessed item relevance, clarity, and cultural appropriateness. Revisions were made based on their recommendations.

A pilot test was conducted among 30 secondary school students in a non-participating school to assess clarity, comprehension, and feasibility. Minor wording adjustments were made to improve readability. Internal consistency reliability was evaluated for multi-item domains (e.g. perception-related items), and Cronbach’s alpha coefficients were within acceptable ranges (>0.70), indicating satisfactory reliability.

### Outcome measures

The primary outcome was the prevalence of the tendency to use e-cigarettes among students aged 13–18 years in Chachoengsao Province, Thailand. In the context of this study, ‘tendency’ was operationalized as early cognitive openness or a tendency toward e-cigarette use, rather than a confirmed behavioral intention.

Data were collected using an anonymous, self-administered online questionnaire. Participants were asked: ‘Do you have thoughts about using electronic cigarettes?’. Responses were dichotomized as ‘no’ or ‘yes.’ A ‘yes’ response was interpreted as indicating a tendency (i.e. cognitive openness) toward future use.

We acknowledge that tendencies, both behavioral and dispositional, are multidimensional constructs that may include intention, willingness, and predisposition factors. Using a single dichotomized item may not fully capture these dimensions and may introduce measurement error. However, this approach was selected to ensure feasibility in a large school-based survey and to minimize respondent burden.

The study did not directly assess current conventional cigarette smoking or e-cigarette use. Given that the sale and importation of e-cigarettes are prohibited in Thailand, directly asking about current use in a school-based setting may increase social desirability bias and underreporting. Therefore, tendency (i.e. cognitive openness or consideration) was used as a proxy indicator of potential future uptake. This construct has been widely used in adolescent tobacco research as an early predictor of smoking initiation.

### Statistical analysis

Survey data were exported from the REDCap system^16^, verified for accuracy and completeness, and analyzed using STATA version 17 (StataCorp LLC, 2022). Descriptive statistics (frequency and percentage) were used to summarize demographic data, associated factors, e-cigarette literacy, and the tendency to use e-cigarettes. Associations between independent variables and the tendency to use e-cigarettes were first examined using univariable logistic regression. Variables with theoretical relevance and/or p<0.20 in univariable analysis were entered into a multivariable logistic regression model. The dependent variable was the tendency to use e-cigarettes (no tendency vs tendency). Independent variables considered in the model included sex, age, GPA, family monthly income, parental education level, parental marital status, living arrangement, perceived family economic status, exposure to smoking, exposure to e-cigarette advertisements, perceived guardian and peer attitudes, admired persons’ e-cigarette use, encouragement to use e-cigarettes, and perceptions regarding stress relief, portability, flavors, accessibility, and social acceptance. Results are reported as adjusted odds ratios (AORs) with 95% confidence intervals (CIs). Statistical significance was set at p<0.05.

## RESULTS

### Characteristics of participants

**Figure 1:**
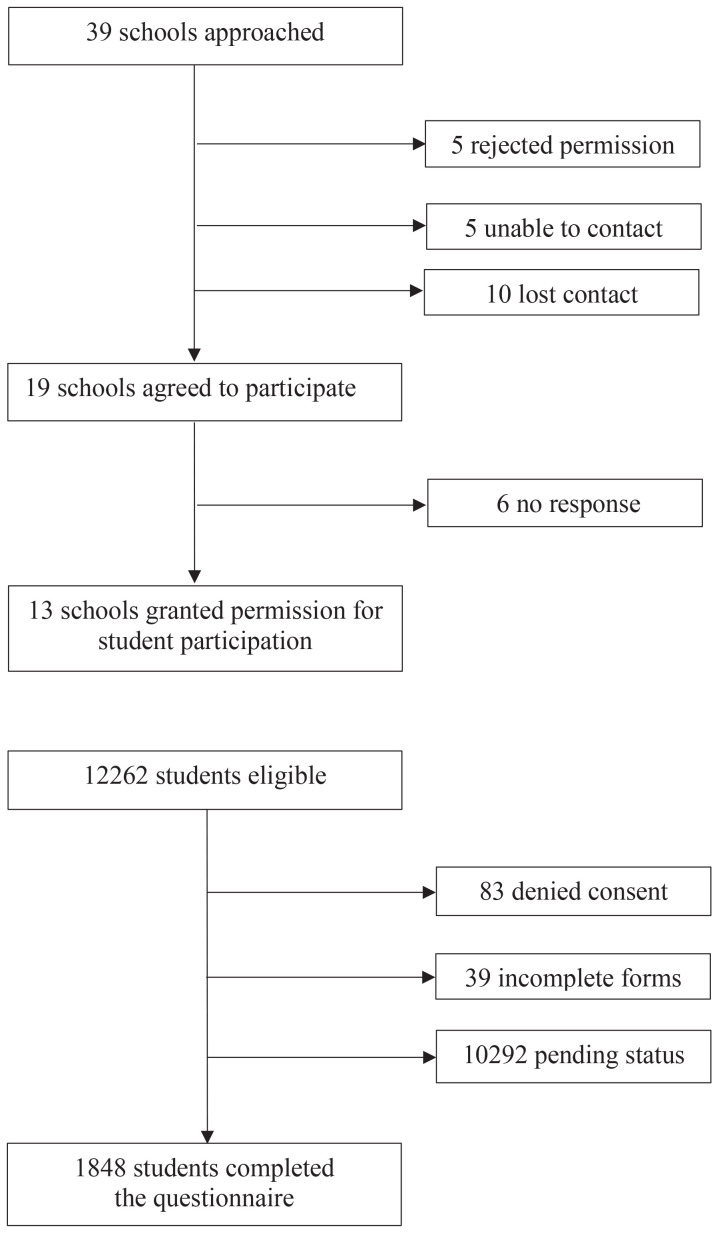
Flowchart of participant recruitment and data collection process

A total of 39 secondary schools in Chachoengsao Province, Thailand, were approached for participation. Nineteen schools agreed to participate, and 1848 students aged 13–18 years completed the online questionnaire (Figure 1). Of these, 54.7% were female, with a mean age of 14.9 ± 1.6 years. The majority of participants were enrolled in lower secondary grades (Grades 7–9), whereas fewer students were from upper secondary grades (Grades 10–12). This distribution contributed to the relatively younger mean age observed in the study sample. Most were Thai (98.7%) and Buddhist (96.1%), and the majority were enrolled in schools under the Office of the Basic Education Commission (90.3%). Approximately one-fourth had a GPA between 3.00 and 3.49, and 37.2% reported a family monthly income of ≥30000 THB. Over half (53.9%) lived with both parents, and 45.9% reported that their parents were married and living together (Table 1).

**Table 1:** Characteristics of secondary school students aged 13–18 years in Chachoengsao Province, Thailand, 2024–2025 (N=1848)

*Characteristics*	*n (%)*
**School affiliation** (N=1839)	
Office of the Basic Education Commission	1660 (90.27)
Private school	71 (3.86)
Municipal School	108 (5.87)
**District** (N=1839)	
Mueang Chachoengsao	686 (37.3)
Plaeng Yao	422 (22.95)
Bang Nam Priao	253 (13.76)
Bang Pakong	176 (9.57)
Khlong Khuean	90 (4.89)
Ban Pho	82 (4.46)
Phanom Sarakham	77 (4.19)
Tha Takiap	53 (2.88)
**Cumulative GPA in recent semester**	
<2.00	148 (8.01)
2.00–2.49	324 (17.53)
2.50–2.99	384 (20.78)
3.00–3.49	514 (27.81)
3.50–4.00	478 (25.87)
**Sex**	
Female	1010 (54.65)
Male	838 (45.35)
**Age** (years), mean ± SD (range: 13–18)	14.91 ± 1.62
**Ethnicity**	
Thai	1823 (98.65)
Other	25 (1.35)
**Religion**	
Buddhist	1776 (96.1)
Muslim	37 (2)
Christian	21 (1.1)
No religion	11 (0.6)
Other	3 (0.16)
**Family monthly income** (THB) (N=1817)	
Median (IQR) (range: 1000–700000)	20000 (10950–30000)
<5000	72 (3.96)
5000–9999	141 (7.76)
10000–19999	529 (29.11)
20000–29999	400 (22.01)
≥30000	675 (37.15)
**Idea of smoking**	
Never	1002 (54.22)
Ever	846 (45.78)
**Idea of drinking alcohol**	
Never	776 (56.93)
Ever	587 (43.07)

GPA: grade point average. IQR: interquartile range. THB: 1000 Thai Baht about US$31.

Regarding behaviors and social environment, 45.8% had ever considered smoking, and 43.1% had considered drinking alcohol. Nearly four in five (79.7%) reported seeing people smoking around them, while 43.5% had been exposed to e-cigarette advertisements. Although 81.1% reported that their parents/guardians disapproved of e-cigarette use, 4.2% indicated that their friends were supportive. About one-third believed that e-cigarette use could enhance social acceptance, and two-thirds cited flavors as a key attraction (Table 2).

**Table 2:** Associated factors influencing e-cigarette use by secondary school students aged 13–18 years in Chachoengsao Province, Thailand, 2024–2025 (N=1848)

*Factors*	*n (%)*
**Paternal education level**	
No formal	61 (3.3)
Primary	357 (19.32)
Lower secondary	436 (23.59)
Upper secondary	448 (24.24)
Vocational certificate (diploma)	196 (10.61)
Higher vocational certificate (advanced diploma)	144 (7.79)
Bachelor’s degree or higher	206 (11.15)
**Maternal education level**	
No formal	47 (2.54)
Primary	268 (14.5)
Lower secondary	438 (23.7)
Upper secondary	519 (28.08)
Vocational certificate (diploma)	175 (9.47)
Higher vocational certificate (advanced diploma)	139 (7.52)
Bachelor’s degree or higher	262 (14.18)
**Marital status of parents**	
Single	49 (2.65)
Married and living together	849 (45.94)
Married but not living together	71 (3.84)
Not married but living together	207 (11.2)
Widowed	31 (1.68)
Divorced/separated/ended relationship	641 (34.69)
**Living arrangement with parents**	
Living with both father and mother	996 (53.9)
Living with father	206 (11.15)
Living with mother	418 (22.62)
Not living with either father or mother	228 (12.34)
**Perception of family economic status**	
Low	151 (8.17)
Moderate	1682 (91.02)
Wealthy	15 (0.81)
**Have you ever seen people around you smoking? If yes, please specify who you have seen**	
Never	375 (20.29)
Yes	1473 (79.71)
**What do you think is your guardian’s attitude toward using e-cigarettes?**	
Opposed or strictly prohibited	1498 (81.06)
Neutral, considers it normal without expressing opinions	341 (18.45)
Supportive or encourages trying it	9 (0.49)
**What do you think are your friends’ attitudes toward using e-cigarettes?**	
Not supportive or strictly prohibited	935 (50.6)
Neutral or no opinion expressed	835 (45.18)
Supportive or encourages trying it	78 (4.22)
**Have you ever seen advertisements for e-cigarettes?**	
Never	1044 (56.49)
Yes	804 (43.51)
**Do the people you admire or regularly follow (on social media or in real life) use e-cigarettes?**	
No	1269 (71.61)
Yes	503 (28.39)
**Have you ever been encouraged to use e-cigarettes? If yes, please specify who encouraged you (optional)**	
Never	1253 (67.8)
Yes	
Parent	19 (0.01)
Teacher/school staff	13 (0.01)
Peer	555 (30.0)
**Do you think that e-cigarettes can help relieve stress if you are feeling stressed?**	
No	656 (35.5)
Yes	1192 (64.5)
**Do you think that the portability of e-cigarettes contributes to their increased usage?**	
No	847 (45.83)
Yes	1001 (54.17)
**Do you think that the variety of scents (pleasant and non-lingering) in e-cigarettes contributes to their increased usage?**	
No	683 (36.96)
Yes	1165 (63.04)
**Do you think that the variety of flavors in e-cigarettes contributes to their increased usage?**	
No	635 (34.36)
Yes	1213 (65.64)
**Do you think that e-cigarettes within the price range 200–3000 Baht are accessible to you?**	
No	933 (50.49)
Yes	915 (49.51)
**Do you think that using e-cigarettes affects your social acceptance among peers or the community you are part of?**	
No	1311 (70.94)
Yes	537 (29.06)

In terms of knowledge and perception, most students (92.5%) recognized that e-cigarettes are harmful, and 85.8% understood that they can cause serious diseases such as coronary artery disease or stroke. However, misconceptions persisted: 36.2% believed that e-cigarettes are less harmful than conventional cigarettes, 29.6% thought they were non-addictive, and 27.1% believed they could help people quit smoking. Although 74.9% were aware that the sale or purchase of e-cigarettes is illegal in Thailand, nearly one-fifth (17.9%) were uncertain (Table 3).

**Table 3:** Knowledge about e-cigarettes among secondary school students aged 13–18 years in Chachoengsao Province, Thailand, 2024–2025 (N=1848)

*Items*	*n (%)*
**E-cigarettes cause harm to the user**	
True	1710 (92.53)
False	41 (2.22)
Not sure	97 (5.25)
**Using e-cigarettes can lead to serious diseases such as coronary artery disease, stroke, or hypertension**	
True	1585 (85.77)
False	46 (2.49)
Not sure	217 (11.74)
**Inhaling vapor from others’ e-cigarette use can harm your health**	
True	1572 (85.06)
False	58 (3.14)
Not sure	218 (11.8)
**Compared to traditional cigarettes, e-cigarettes are less harmful**	
True	668 (36.15)
False	753 (40.75)
Not sure	427 (23.11)
**Compared to traditional cigarettes, e-cigarettes leave fewer residues**	
True	591 (31.98)
False	610 (33.01)
Not sure	647 (35.01)
**Using e-cigarettes does not lead to addiction**	
True	547 (29.6)
False	941 (50.92)
Not sure	360 (19.48)
**Using e-cigarettes can help you quit smoking traditional cigarettes**	
True	500 (27.06)
False	793 (42.91)
Not sure	555 (30.03)
**It is easy to quit e-cigarette addiction**	
True	432 (23.38)
False	909 (49.19)
Not sure	507 (27.44)
**Buying or selling e-cigarettes is illegal**	
True	1385 (74.95)
False	133 (7.2)
Not sure	330 (17.86)

Overall, 17.6% of respondents (n=326) reported a tendency to use e-cigarettes, while 82.4% (n=1522) reported no such inclination. Multivariate logistic regression identified several factors that were significantly associated with the tendency to use e-cigarettes (Table 4). Female students were more likely than males to report a tendency to use e-cigarettes (AOR=1.66; 95% CI: 1.20–2.30; p=0.002). Students from households with a monthly income below 5000 THB had higher odds compared with those from households earning 20000–29999 THB (AOR=2.81; 95% CI: 1.26–6.25; p=0.011). Having ever considered smoking was the strongest predictor of tendency to use e-cigarettes among measured variables(AOR=6.71; 95% CI: 4.59–9.82; p<0.001), and those who had considered drinking alcohol also had a higher likelihood of tendency to e-cigarette use (AOR=1.71; 95% CI: 1.16–2.54; p=0.007). Students whose friends had neutral attitudes toward vaping had higher odds (AOR=1.42; 95% CI: 1.01–2.01; p=0.043). Students who had ever been encouraged to use e-cigarettes had significantly higher odds (AOR=1.95; 95% CI: 1.39–2.74; p<0.001). Students who believed that e-cigarettes cannot help relieving stress when feeling stressed had significantly higher odds (AOR=2.34; 95% CI: 1.71–3.19; p<0.001). Students who believed that using e-cigarettes affects their social acceptance among peers or within their community were more likely to use e-cigarettes (AOR=1.75; 95% CI: 1.27–2.41; p=0.001). Knowledge-related misconceptions were also associated with tendency: being unsure whether e-cigarettes could cause serious diseases (AOR=1.75; 95% CI: 1.04–2.97; p= 0.036), believing that inhaling secondhand vapor is harmless (AOR=3.68; 95% CI: 1.57–8.64; p=0.003), and believing that e-cigarettes help quit smoking (AOR=1.93; 95% CI: 1.29–2.89; p=0.001).

**Table 4:** Multivariable logistic regression analysis of factors associated with tendency to use e-cigarettes among secondary school students aged 13–18 years in Chachoengsao Province, Thailand, 2024–2025 (N=1848)

*Variables*	*No tendency to use e-cigarettes n (%)*	*Tendency to use e-cigarettes n (%)*	*AOR*	*95% CI*	*p*
**Sex**					
Male (ref.)	693 (82.7)	145 (17.3)	1		
Female	829 (82.1)	181 (17.9)	1.66	1.20–2.30	0.002
**Family monthly income** (THB)					
<5000	55 (76.4)	17 (23.6)	2.81	1.26–6.25	0.011
5000–9999	111 (78.7)	30 (21.3)	1.56	0.84–2.91	0.157
10000–19999	431 (81.5)	98 (18.5)	1.24	0.80–1.91	0.344
20000–29999 (ref.)	339 (84.8)	61 (15.2)	1		
≥30000	566 (84.0)	108 (16.0)	1.05	0.68–1.61	0.834
**Consideration of smoking**					
Never (ref.)	1244 (93.7)	84 (6.3)	1		
Ever	278 (53.5)	242 (46.5)	6.71	4.59–9.82	<0.001
**Consideration of drinking alcohol**					
Never (ref.)	935 (93.3)	67 (6.7)	1		
Ever	587 (69.4)	259 (30.6)	1.71	1.16–2.54	0.007
**What do you think are your friends’ attitudes toward using e-cigarettes?**					
Opposed or strictly prohibited (ref.)	845 (90.4)	90 (9.6)	1		
Neutral or no opinion expressed	626 (75.0)	209 (25.0)	1.42	1.01–2.01	0.043
Supportive or encourage trying it	51 (65.4)	27 (34.6)	1.17	0.60–2.31	0.641
**Have you ever been encouraged to use e-cigarettes?**					
No (ref.)	1138 (90.8)	115 (9.2)	1		
Yes	376 (64.1)	211 (35.9)	1.95	1.39–2.74	<0.001
**Do you think that e-cigarettes can help relieve stress if you are feeling stressed?**					
No	460 (70.1)	196 (29.9)	2.34	1.71–3.19	<0.001
Yes (ref.)	1062 (89.1)	130 (10.9)	1		
**Do you think that using e-cigarettes affects your social acceptance among peers or the community you are part of?**					
No (ref.)	1156 (88.2)	155 (11.8)	1		
Yes	366 (68.2)	171 (31.8)	1.75	1.27–2.41	0.001
**Using e-cigarettes can lead to serious diseases such as coronary artery disease, stroke, or hypertension**					
True (ref.)	1318 (83.2)	267 (16.8)	1		
False	37 (80.4)	9 (19.6)	1.41	0.48–4.18	0.532
Not sure	167 (77.0)	50 (23.0)	1.75	1.04–2.97	0.036
**Inhaling vapor from others’ e-cigarette use can harm your health**					
True (ref.)	1312 (83.5)	260 (16.5)	1		
False	39 (67.2)	19 (32.8)	3.68	1.57–8.64	0.003
Not sure	171 (78.4)	47 (21.6)	0.85	0.49–1.49	0.577
**Using e-cigarettes can help you quit smoking traditional cigarettes**					
True	373 (74.6)	127 (25.4)	1.93	1.29–2.89	0.001
False (ref.)	679 (85.6)	114 (14.4)	1		
Not sure	470 (84.7)	85 (15.3)	1.14	0.74–1.77	0.546

AOR: adjusted odds ratio. Model adjusted for all variables presented in the table. THB: 1000 Thai Baht about US$31.

## DISCUSSION

This study found that a considerable proportion of secondary school students demonstrated tendency to use e-cigarette (17.6%). Because tendency reflects cognitive openness rather than actual behavior, it should not be directly compared with previously reported prevalence estimates of ever or current e-cigarette use in Thailand. Instead, this finding highlights the presence of a substantial subgroup of adolescents who may be cognitively vulnerable to future experimentation. While prior national data reported lower levels of ever and current use in 2021, our results suggest that potential risk for initiation remains an important public health concern, particularly in contexts where e-cigarettes are prohibited yet remain accessible through online and informal markets. Similar concerns regarding adolescent vulnerability have been described in other countries, such as the United States and the United Kingdom^4,17^. The increasing prevalence highlights a widening public-health concern, particularly within contexts where e-cigarettes are banned yet remain accessible through online and informal markets^13^.

Consistent with previous research, the current study identified several behavioral and psychosocial factors associated with the tendency to use e-cigarettes. Students who had previously considered smoking were more than six times as likely to report an intention to use e-cigarettes, emphasizing the shared behavioral pathway between conventional tobacco and vaping initiation. Similarly, having ever considered drinking alcohol was associated with greater odds of tendency to e-cigarette use, consistent with evidence that multiple health-risk behaviors cluster during adolescence. Peer influence also played a significant role: students whose friends were neutral toward vaping were more likely to report tendency, consistent with findings that social networks and peer norms reinforce experimentation with e-cigarettes^18^. In addition, direct social encouragement emerged as a significant factor. Students who had ever been encouraged to use e-cigarettes had nearly twice the odds of reporting an intention to use, highlighting the strong influence of interpersonal dynamics during adolescence. This finding suggests that, beyond general peer norms, explicit invitations or persuasive behaviors from friends may serve as proximal triggers for experimentation.

Perceptions related to social image and stress management were also significantly associated with tendency. Students who believed that using e-cigarettes affects their social acceptance among peers or within their community were more likely to report intention to use, suggesting that vaping may be perceived as a strategy to enhance social belonging or status. Furthermore, students who believed that e-cigarettes cannot help relieving stress when feeling stressed, had higher odds of intention to use. Although this finding may appear counterintuitive, it may reflect complex and overlapping cognitive patterns among adolescents. Perceptions about stress relief may not represent the primary motivation for experimentation. Instead, adolescents may express cognitive openness toward e-cigarettes due to peer norms, curiosity, or social identity factors, regardless of their beliefs about stress management. After adjustment for correlated psychosocial variables in the multivariable model, the direction of association may reflect residual confounding or interaction effects rather than a direct causal pathway.

An interesting finding was that students from lower income households (<5000 THB per month) had a higher likelihood of expressing a tendency to use e-cigarettes. This contrasts with studies from Western countries, where higher socioeconomic status has been linked to greater vaping access^12^. One possible explanation is that adolescents from lower income backgrounds may experience higher stress levels or weaker parental supervision, increasing vulnerability to peer pressure and risk-taking behaviors. Another possibility is the availability of inexpensive or counterfeit vaping products in informal markets, which lowers financial barriers to use^1^. These findings underscore the importance of addressing socioeconomic disparities in adolescent health promotion programs^14^.

Knowledge and perception were also key determinants. Students with misconceptions – such as believing that secondhand vapor is harmless or that e-cigarettes can help people quit smoking – were more likely to express an intention to use them. These misunderstandings persist despite scientific evidence demonstrating that aerosols from e-cigarettes contain nicotine and toxic substances associated with respiratory and cardiovascular damage^6,7^. Misbeliefs about stress relief and social acceptance reflect how targeted marketing and peer communication distort adolescents’ risk perceptions. Improving health literacy and fostering critical understanding of vaping-related harms are, therefore, essential components of youth prevention programs^6,7^.

The belief that e-cigarettes can help people quit smoking may reflect broader harm-reduction perceptions rather than a literal intention to use them for smoking cessation. Adolescents who endorse this belief may perceive e-cigarettes as medically acceptable or less harmful alternatives to conventional cigarettes. Such reduced risk perception may lower psychological barriers to experimentation and increase cognitive openness to use, even among non-smokers. Therefore, this association likely reflects diminished perceived harm rather than a direct cessation-related motive.

### Limitations

The large sample size and inclusion of diverse behavioral and knowledge variables enhance the validity of the findings. However, certain limitations should be noted. The cross-sectional design precludes causal inference. Furthermore, the use of self-reported measures may have resulted in recall and social desirability bias, with the possibility of non-differential misclassification of key variables. School participation was voluntary, and certain districts were underrepresented, which may restrict the external validity of the findings. Although behavioral and socio-economic factors were included in the model, residual confounding due to unmeasured variables – such as parental smoking, mental health conditions, or exposure to online marketing – cannot be excluded. These challenges are common in adolescent behavioral research^12,18^. Furthermore, the study did not include direct measures of current cigarette or e-cigarette use. While tendency is a validated predictor of future initiation, it does not necessarily equate to actual behavior. Therefore, the findings should be interpreted as reflecting early cognitive vulnerability rather than established tobacco use patterns.

The relatively younger age distribution of participants should be considered when interpreting the findings. As most respondents were enrolled in lower secondary grades, the results may primarily reflect early adolescence rather than older adolescents. Behavioral patterns and risk exposure may differ among upper secondary students. Therefore, caution is warranted when generalizing the findings to all adolescents aged 13–18 years in Thailand.

### Implications

Despite these limitations, this study provides important baseline evidence on adolescent tendency to e-cigarette use in a rural Thai setting. The findings indicate that conventional health education and regulatory approaches alone may be insufficient to address this growing public health concern. Given the significant influence of peer norms and safety-related misconceptions identified in this study, these findings highlight the importance of addressing social influences and risk perceptions in adolescent populations. Efforts to enhance critical awareness of the health, social, and economic consequences of smoking and vaping may be relevant in contexts where e-cigarette use is becoming normalized within school communities. Future research employing longitudinal designs is needed to clarify temporal relationships and further examine these associations, particularly among high-risk groups identified in this study.

## CONCLUSIONS

This cross-sectional study found that nearly one in six secondary school students in Chachoengsao Province reported a tendency to e-cigarette use despite the national ban. Being female, lower household income, prior consideration of smoking and alcohol use, peer influence, and safety-related misconceptions were significantly associated with tendency.

These findings highlight the importance of addressing peer norms, cognitive openness to substance use, and vaping-related misconceptions in adolescent populations. Given the cross-sectional design, causal inferences cannot be drawn. Longitudinal research is warranted to further clarify temporal relationships and to inform evidence-based prevention strategies.

## Supplementary Material



## Data Availability

The data supporting this research are available from the authors on reasonable request.
